# Endogenous dynamics of denunciation: Evidence from an inquisitorial trial

**DOI:** 10.1093/pnasnexus/pgae340

**Published:** 2024-08-21

**Authors:** José Luis Estévez, Davor Salihović, Stoyan V Sgourev

**Affiliations:** Department of Economic and Social History, University of Helsinki, Snellmaninkatu 14 A, 00014 Helsinki, Finland; Population Research Institute, Väestöliitto, Kalevankatu 16, 00101 Helsinki, Finland; Department of History, University of Antwerp, Sint-Jacobsmarkt 13, 2000 Antwerp, Belgium; Department of Organizational Studies and HR Management, EM Normandie Business School, 30-32 Rue Henri Barbusse, 92110 Clichy, France

**Keywords:** denunciation, Inquisition, endogenous dynamics, social control, social networks

## Abstract

We develop an endogenous approach to the practice of denunciation, as an alternative to exogenous historical and sociological accounts. It analyzes denunciation as a response to increasing pressure, which in turn increases pressure on social contacts. The research context is the trial of Waldensians in Giaveno, Italy, in 1335, headed by the inquisitor Alberto de Castellario. A dynamic network actor model attests that coercive pressure not only raises the rate of denunciation but also compels denouncers to implicate individuals who are socially closer to them. We find that coercive pressure starts yielding diminishing returns relatively quickly, with the degree of redundancy of information escalating as a result of preferential attachment, increasingly targeting those already denounced by others, publicly announced suspects, and those having absconded from the trial.

**Significance Statement**

The analysis articulates relational mechanisms whereby repressive authorities exercise control over citizens by infiltrating social networks, but also intrinsic limitations to their application. Its historical significance lies in documenting the preoccupation with efficiency rather than effectiveness of the inquisitorial legal process, providing new insights into why relatively successful local crackdowns by the medieval Inquisition did not manage to prevent the global dissemination of religious dissent. Its contemporary relevance is in showing that endogenous forces restrain the effectiveness of coercive pressure, requiring its reinforcement in order to sustain denunciation: a dynamic widely observed in current authoritarian regimes. Abundant scholarship documents the emergence of social networks; we direct attention instead to how social connections break down.

## Introduction

In the aftermath of the “Red Scare” in 1950s Hollywood, Orson Welles observed forlornly that “We are very few in our generation who have not given other people's names. That is terrible. It can never be undone (in 1).” His words testify to a paradox: the provision to the authorities of potentially discrediting information on contacts is a morally reprehensible act that is, nevertheless, common in social life and history. The limited surveillance ability of the state fosters reliance on informers and ordinary citizens, conveying privately collected information to the authorities (2, 3). Democratic governments may embrace practices of denunciation in the service of political (e.g. 4) or economic objectives (e.g. 5), but these play a more fundamental role in authoritarian regimes as a means of repression of dissent. Consider the decision of the Chinese government to provide monetary rewards for tip-offs on issues of national security (6), or the legislation promulgated by the Russian government, encouraging its citizens to report behavior or online comments that manifest opposition to the war in Ukraine (7). Denunciation is a key instrument in the intensification of social control over recalcitrant segments of the population, involving the application of physical force to obstruct collective action (8), and the use of advanced technology for purposes of public surveillance (9).

The recognition that denunciation often occurs in response to increasing pressure, which in turn serves to increase pressure on social contacts, is the starting point of a study of denunciation as a process, rather than as an individual act or predisposition. The value of this analytical approach is twofold. First, it sheds light on the relational mechanisms whereby repressive authorities exercise control over citizens by infiltrating hard-to-reach sections of social networks. Second, it provides an endogenous framework for the study of denunciation that constitutes an alternative to historical and sociological frameworks that are exogenous in nature. Both historical research and sociological research tend to examine denunciation in terms of stable institutional features and motivations associated with them, comparing across historical periods or institutional regimes (e.g. 10–13). We, instead, prioritize situational factors and dynamic change, examining the act of denunciation in the process of adapting to intensifying coercive pressure. This approach adopts the recommendation of White (14) to explore how “direct and indirect chains of ties and their stories are generated in an endogenous process, without need for the analyst to call on attributes or ideology,” as is customary in dominant accounts. In theoretical terms, it builds on the “processual” approach in sociology (15, 16). We share two of its key assumptions. One is its emphasis on events as the unit of analysis. Understanding individual actions, such as reporting a neighbor or a relative to the authorities, requires identifying sequences of events and the mechanisms linking them together (16). The other is the necessity to combine sociological generalization with historical attention to timing and order. The social order emerges from sequences of actions by particular actors at particular times in particular places (15).

The particular historical event that we analyze is a 14th-century inquisitorial trial. This is an appropriate context for the dynamic analysis of denunciation for three reasons. First, the arrival of an inquisitor was a highly disruptive event, unsettling local customs and social networks (e.g. 17, 18). Second, a trial features a methodologically advantageous combination of a fixed location, well-defined formal stages, and a sufficient duration (from several weeks to a few months) to facilitate observation of the endogenous chains of ties (14). Third, an inquisitorial trial featured both cooperative and coercive practices of collection of information.

A dynamic network actor analysis (19) of the rate and target of denunciation throughout the trial allows us to estimate the utility of coercive pressure in extracting information, but also identify its limitations. The results attest that coercive pressure is efficient in extracting information but starts yielding diminishing returns relatively quickly. This is a consequence of preferential attachment (20), as individuals increasingly target those already denounced by others, publicly announced suspects, and those having absconded from the trial. Revealing the emphasis on efficiency rather than effectiveness of the inquisitorial process contributes to better understanding why successful local control by the medieval Inquisition did not manage to prevent the global diffusion of religious dissent.

## The medieval Inquisition

The featured trial was only a link in a long-term historical process of combating the spread of what the Catholic Church considered “new and false” beliefs, contrary to Holy Scripture (18). The category of “heresy” was constructed by using a set of rhetorical and cultural tropes to articulate its Satanic origin and “infectious” nature (21). That heresy was a source of “infection” of faithful Christians was viewed as justifying the pursuit, interrogation, and exclusion of heretics from the community (22). The legal framework of the Inquisition developed in a series of decrees during the late 12th and the 13th centuries. Antiheretical trials gradually adopted the procedure introduced by Pope Innocent III in the early 13th century for ecclesiastical courts. It allowed the judge to present charges against a defendant ex officio, without having to rely on accusations by private parties (23). An inquisitor was appointed by the Pope for a particular area, with the authority to detect suspects, establish their guilt through interrogation, secure an abjuration of heresy from the suspect, and pronounce a sentence (if found guilty) (18).

The medieval Inquisition never constituted a centralized institution, coordinating the work of inquisitors (24). Inquisitors operated pragmatically in adapting to local conditions, forming partnerships with bishops and secular authorities (25). A trial typically started with a period of voluntary denunciation, interviewing local clergy, willing to assist in ensuring the purity of the faith by sanctioning unorthodox beliefs. This was followed by a “period of grace”: those who voluntarily presented themselves within a specified period and told the truth about themselves and others were promised leniency (26). After the expiration of the grace period, inquisitors could turn the knob of pressure, trying to cut individuals out of the social networks in which they were embedded (17). To that end, they made use of a set of techniques, such as issuing summonses, requesting a second deposition, incarceration, and torture. A description of interrogatory techniques is provided in the manuals of the inquisitors Bernard Gui (around 1324) and Nicholas Eymerich (1376) (see 18).

Inquisitors were entitled to start an investigation on the evidence of a single witness but were generally reluctant to convict unless this evidence could be corroborated by others (17). This required the collection of extensive information, such as by following up on contacts revealed by those who had just confessed. Every confession required disclosure of other heretics known to the person (18), which facilitated the intensification of coercive pressure and contributed to a state of anxiety, as individuals were unsure as to the behavior of others. The inquisitor's attempts to pressure deponents were frequently met with resistance, such as threatening those who might testify against them or concluding pacts to conceal incriminating evidence, typically based on kin ties (17).

The outcome of a trial was not predetermined, as it depended on how skillfully an inquisitor managed to apply sustained pressure on suspects and the degree to which suspects self-organized. They had to choose a defensive strategy, such as ostracism, open hostility, flight, collaboration with the inquisitor, or appeal to others for help, and then decide whether to disrupt networks at the family, neighborhood, or village level (27). They could preserve the cohesion of the family by implicating fellow villagers, refuse any form of collaboration, or collaborate by targeted disruption of the extended family.

## The trial of the Waldensians in Giaveno (1335)

The Waldensians were founded by Vaudès, a wealthy resident of Lyon, France, who gave away his possessions around 1170. They were an ascetic religious community, based on the strict observance of the gospel, preaching, and poverty. Originally part of the Church (21), they were declared heretics by 1215 because of their unwillingness to recognize the power of local bishops. The trial of the Waldensians in Giaveno by the inquisitor Alberto de Castellario in early 1335 is recorded in a register kept at the Archivio Generale dell’Ordine dei Predicatori in Rome as MS II.64, ff 1r-111v. We use the modern edition published in Merlo (28). The register contains various deeds, including notes on summonses, summonses issued for specific people, reports by officers, notes from interrogations, abjurations, culpae of individuals (a final listing of misdeeds), and sentences.

Alberto de Castellario was a Dominican friar, likely born in Cuneo. His dispatch to Giaveno was motivated by his local origin and personal experience with the Waldensians. The trial started on January 20 1335 and ended on February 26. The first 10 days de Castellario interviewed local priests and laypeople. On January 29, he called upon those with knowledge of heretics to appear before him within 3 days (the period of grace). This resulted in a few denunciations, incriminating what appeared to be far-off social connections. On that basis, de Castellario issued summonses on February 2 against 12 people. Nine of them appeared before him the next day, denying any connection to the Waldensians. On February 4, Iohannes Gauterii was summoned. He likewise denied any heretical activity. The inquisitor decided to torture him, which resulted in Gauterii giving 47 names on February 4 and 24 on February 5 (65 unique names altogether). His testimony provided more than a quarter of all names revealed to the inquisitor by the end of the trial.

Following Gauterii's deposition, de Castellario issued summonses for 13 people on February 6, all but one of them appearing for the first time. They provided no names. Dissatisfied with the results, the inquisitor decided to torture Stephanus Vet, who had been deposed before but had denied involvement. Vet cracked, providing 13 names on the first day, and 11 more on the following one. This scenario was repeated with Bernardus de Rosseto: subjected to torture, he provided 13 names. February 4 to 7 marked the turning point in the trial. The illicit network started to unravel and the collective mood changed. On February 9, Palmerius Goytrati re-appeared before the inquisitor “with refreshed memory” to provide information (no record of him being tortured). As Merlo (28) notes, from February 9 all those appearing before the inquisitor seem to have regained their memory, providing names.

Fear of torture likely contributed to the sudden change, even though torture was sparingly used: the notary recorded only four instances throughout the trial. Another factor was the realization by many deponents that they were liable to an accusation of perjury, having previously denied any involvement. However, deponents were likely reacting not only to the actions of the inquisitor but also to those of their peers. Conditions of high uncertainty can lead to the alignment of individual decisions (29). Alignment is “behavioral” in nature, when individuals make a decision by observing the number of those who choose an option (30), or “inferential,” derived from tacitly coordinated inferences of the most plausible outcome (29). These inferences are based on information about the preferences of others, publicly available information, or the behavior of prominent individuals. To understand how individual decisions aggregated, we subjected the denunciations observed during the trial to a formal analysis.

## Results

### Macro-level patterns

**Fig. 1.**
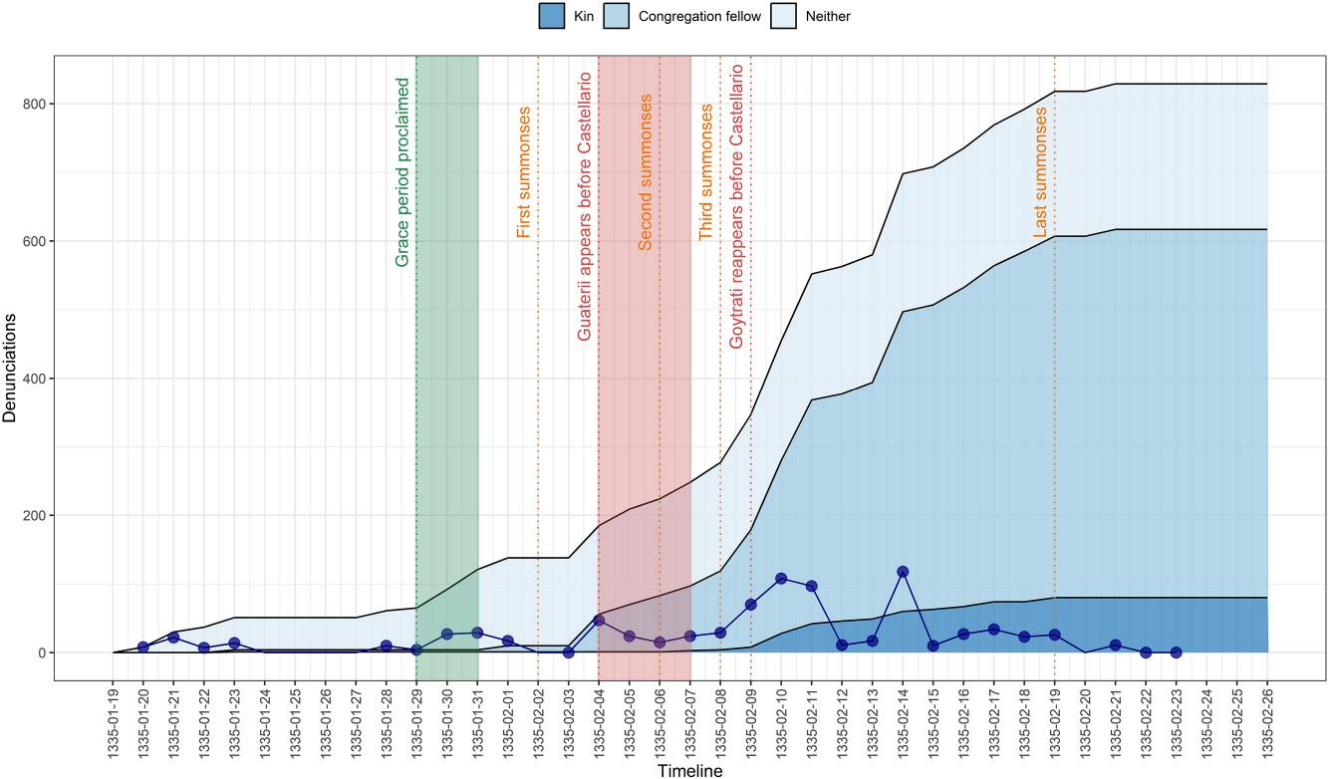
Denunciations during the trial. The connected dots at the bottom of the graph represent the number of denunciations reported to the inquisitor on each day. The cumulative number of denunciations up to a given day is shown in the background, categorized by the relationship between the denouncers and the denounced: kin members (dark tone), congregation fellows (mid tone), or neither of the two (light tone).

We start by plotting the denunciations reported to the inquisitor per day (see Fig. 1). The connected dots at the bottom of the graph represent the daily count of denunciations. In addition, key events that potentially influenced the number of denunciations are highlighted. These events include the grace period (January 29 to January 31), public summonses (February 2, 6, 8 and 19), the torture of Gauterii, Vet, and Rosetto (February 4 to February 7), and Goytrati's redeposition (February 9). In the background is a cumulative plot that categorizes the denunciations based on the relationship between the denouncer and the denounced. These relationships are divided into three categories: kin member, congregation fellow in the heretic network, or a fellow villager (no social connection).

Figure 1 illustrates the shift in denunciation target as the trial progressed and the pressure on deponents increased. Almost all denunciations in the first 2 weeks targeted individuals who were neither congregation fellows nor kin members. By way of the torture and confession of Gauterii, the inquisitor managed to infiltrate the heretic network, with denunciations increasingly targeting congregation fellows. As the denunciation rate escalated, deponents started denouncing kin members. The temporal pattern attests that the Giaveno residents shielded congregation fellows and kin members until they thought it untenable to continue doing so. By the end of the trial, 537 out of the 829 denunciations (64.8%) targeted congregation fellows and 80 (9.7%)—kin members.

**Fig. 2.**
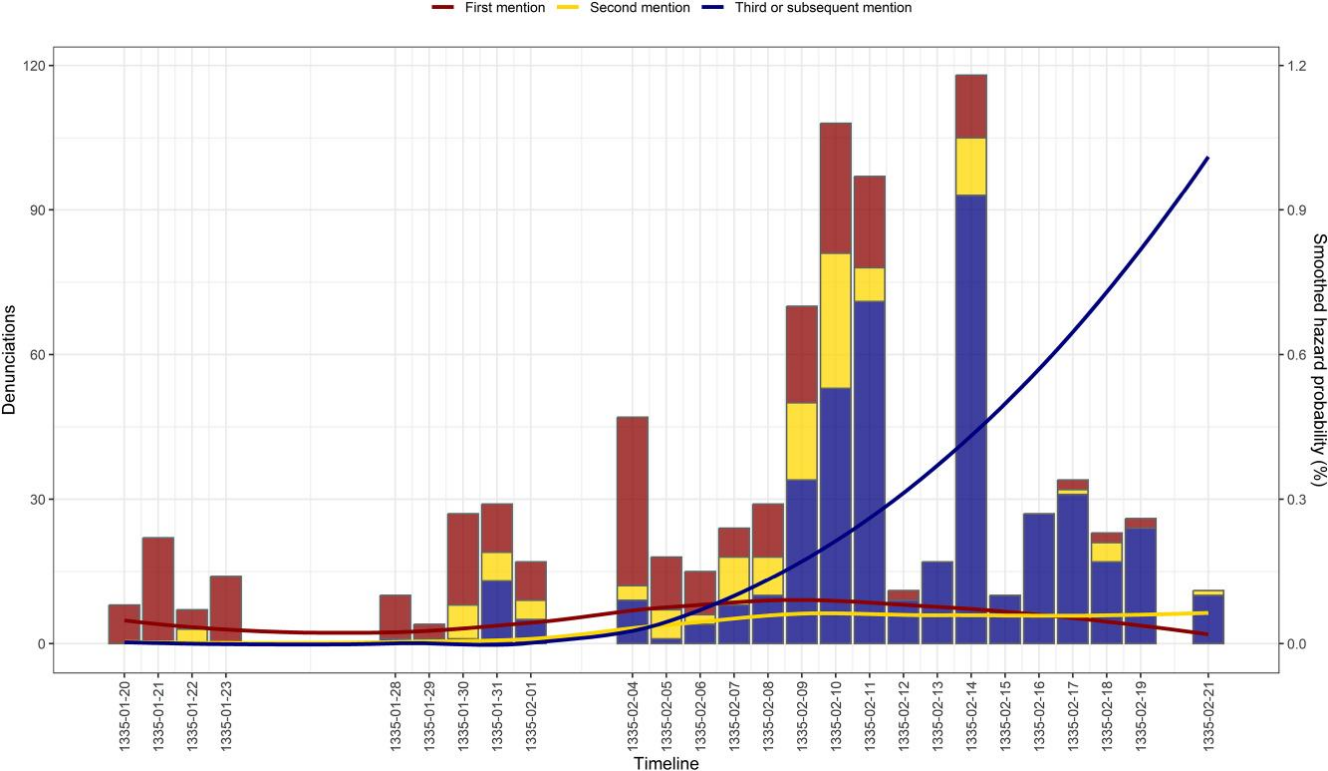
Degree of redundancy in denunciation. The bars represent the number of denunciations reported to the inquisitor, categorized by whether the name of the person reported was mentioned for the first time (dark red), second time (gold), and third or subsequent time (navy blue). The lines depict the hazard probabilities of observing a first, second, third, or subsequent mention, based on the testimony of the 110 deponents and the possibility of reporting any of the 239 individuals (excluding the deponent) identified as heretics to the inquisitor. All ties that are possible but not reported are censored with a deponent's (second) deposition. Values have been smoothed using the LOESS algorithm to highlight the overall pattern.

It appears that the increasing coercive pressure led to shifts in the rate and target of denunciations, but that the denunciation rate leveled off in the last week of the trial. For additional insight, we categorized denunciations based on whether the names were new to the inquisitor or had already been reported by others. Figure 2 yields two important observations: first, the escalating number of denunciations was largely due to denunciations of those already denounced, and second, the names circulating in the last days of the trial were largely familiar to the inquisitor. By the time of Goytrati's redeposition, the tendency for recycling names was tangible. It is likely that the inquisitor brought an end to the trial as a result of the realization of the excessive redundancy of information.

**Fig. 3.**
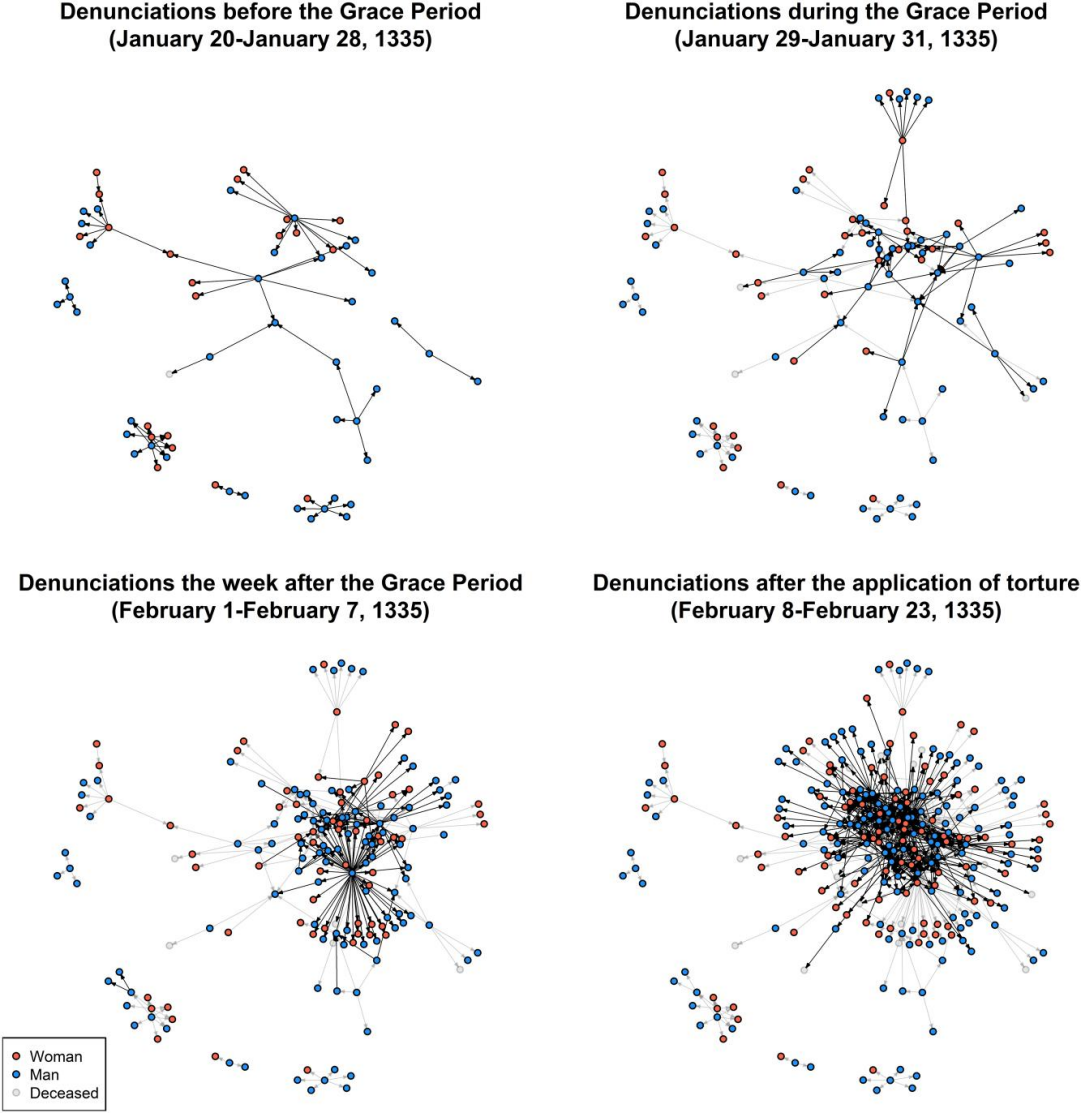
Graph visualizations showing the evolution of the denunciation network at various points during the trial. A directed tie from node *i* to node *j* indicates that *j* was reported as a heretic in *i* ’s deposition(s). Only ties and nodes reported up to the specified time are displayed. Nodes are colored based on gender (red for women, blue for men) and biological state (light gray for deceased individuals). Black ties represent denunciations reported within the specified time frame, while gray ties represent those reported before it.

A bird's-eye view of this process is provided by four snapshots of the evolution of the denunciation network (Fig. 3). In these graphs, each tie represents a denunciation reported to the inquisitor, originating from the deponent and pointing toward the person identified as a heretic. Early denunciations are relatively scattered, allowing the inquisitor a choice of segment to explore. The grace period did not yield many names but provided sufficient information for a more targeted approach. The third graph illustrates the deepening penetration of the clandestine network. The last graph depicts the network at the trial's conclusion. The four snapshots attest that the inquisitor focused on a specific segment of the early network, systematically exploring it until it appeared exhausted, as displayed in the redundancy of information.

### Micro-level patterns

**Fig. 4.**
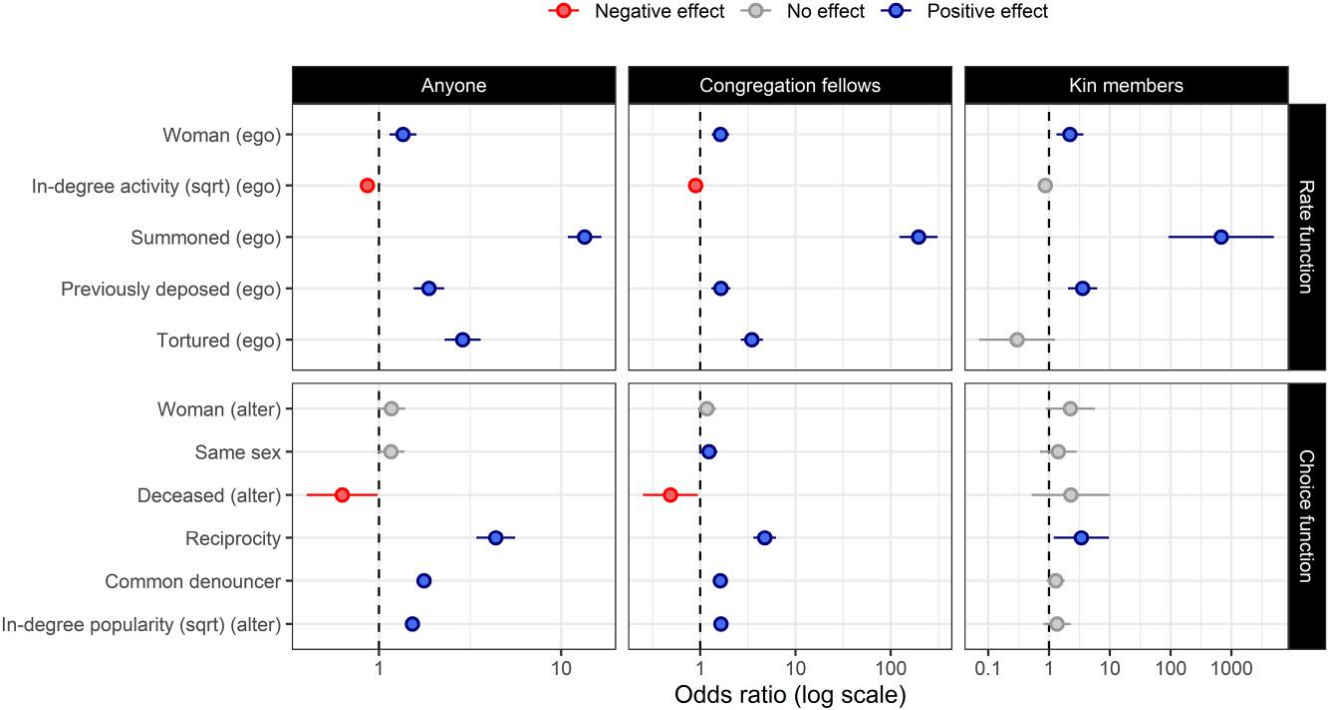
ORs for all the denunciations reported to the inquisitor (left-hand panels), for denunciations targeting congregation fellows (central panels), and for denunciations targeting kin members (right-hand panels). The top panels show values related to the rate function. The bottom panels display values related to the choice function. The dashed vertical line indicates null effect (OR = 1). Values to the left of this line indicate a lower tendency for denunciations to originate from an individual possessing the attribute in question (for the rate function), or a reduced tendency for individuals possessing that trait to be named (for the choice function). Values to the right of this line indicate a greater tendency for denunciations to originate from an individual possessing the attribute in question (for the rate function), or an increased tendency for individuals possessing that trait to be named (for the choice function). In estimating the rate functions, individuals who never deposed were treated as nonpresent. Thus, our analysis is based on the comparison of the 110 deponents. For estimating the choice function, deponents could potentially select from any of the 239 individuals (excluding the deponent) reported as heretics to the inquisitor. When estimating denunciations directed at family members, the choice set is limited to those identified as family members of the denouncer. Statistical significance is captured using two-tailed 95% CI and indicated with either red or blue color, depending on the direction of the effect. Intercepts have been omitted. For results in table form, including goodness-of-fit statistics, see SI Appendix Section B.

To identify micro-level behavioral mechanisms responsible for the observed aggregated patterns, we applied a dynamic network actor analysis (19). Three models were estimated: a first model for all denunciations, regardless of the target; a second model for denunciations directed at congregation fellows; and a third model for denunciations aimed at kin members only (see Fig. 4).

The first two models reveal similar patterns. Exposed to the same level of pressure, women were more likely to denounce than men. It was common for denunciations to originate from someone previously denounced by others (in-degree activity, see Table S2) and then summoned to appear before the inquisitor. All models indicate a significant positive effect for having been previously deposed, stronger when the target is a family member: the odds ratio (OR) for denunciations directed to a congregation fellow is 1.65 (95% CI 1.31–2.08), compared to 3.56 (95% CI 2.05–6.19) when the target is a family member. Deponents could either volunteer or be called for a second deposition. As a rule, the pressure on a deponent was much greater during the second deposition, as the need for reappearance implied a problem with the initial deposition and the looming threat of perjury. Faced with increasing pressure, a deponent is likely to have felt compelled to disclose more by naming a kin member. Torture also had a positive effect, with an OR of 2.88 (95% CI 2.29–3.61), though no effect was found for torture in denunciations of kin members. With only four individuals tortured (one naming two family members), however, the data may be insufficient to estimate this effect accurately. Overall, results confirm that testimonies adapted to the degree of coercive pressure and that naming a kin member was a measure of last resort.

The models provide additional insight on the target of denunciations. We identified a tendency for deponents to name someone who had previously implicated them (reciprocity) or someone who had been named by a mutual denouncer. The formation of reciprocal and transitive relationships may have been coordinated, resulting from collective efforts to shift blame (17, 26), but may have also arisen spontaneously, reinforced by inferences and mutual observation of people who knew each other (29, 30). Additionally, the impact of received denunciations (target) reveals that deponents were inclined to denounce someone already denounced by others. Overall, these effects attest to the challenge faced by the inquisitor in avoiding redundancies and uncovering “new” names. But if deponents were likely to “pile on” by implicating an already denounced fellow villager or congregation member, they did not exhibit the same tendency regarding kin members. This can be interpreted as an attempt to protect a kin member by shifting blame onto the group.

The observed concentration of blame around a few individuals, such as Goytrati (24 denunciations) or Gauterii (22 denunciations), was largely due to their informal status, as indicated by the very high correlation between the eigenvector centrality (31) in the heretical network and their eventual in-degree in the denunciation network (*ρ* = 0.88). However, we noticed that the person who attracted by far the most denunciations (a total of 44) was Villelmus de Oddo, who had absconded from the trial, leaving the village. Another one who absconded was Marguerita Borsseta, most frequently denounced as the “initiator into heresy” (12 times, compared with only 4 for de Oddo or Gauterii)—a more severe charge than hosting or attending a congregation. We included a variable capturing the five known cases of absconding from the trial. The results confirm that, over and above other predictors, absconding inflated the number of denunciations. Absconding reduced the concern with eventual punishment and ensured that the person would not denounce others to the Inquisitor, making him or her a convenient target. Results attest that denunciations reflected informal prominence and reveal an endogenous dynamic of “piling on,” based on public information about those who have been summoned (OR = 1.79 [95% CI 1.44–2.22]) and/or have absconded (OR = 1.66 [95% CI 1.24–2.21]) (see Table 1 and SI Appendix Section C). A substantive implication of the observed dynamic is that the centrality of key individuals is inflated in the count of denunciations and that accounts of heretic networks as heavily centralized (32), based on inquisitorial records, may be overstated.

**Table 1.** OR of denunciation.

	Denunciation (anyone) (1)		Denunciation (anyone) (2)	
	OR	95% CI	OR	95% CI
*Rate function*				
Intercept	0.05	(0.04–0.06) ***	0.05	(0.04–0.06) ***
Woman (ego)	1.35	(1.14–1.60) ***	1.35	(1.14–1.60) ***
In-degree activity (ego) (sqrt)	0.86	(0.80–0.92) ***	0.86	(0.80–0.92) ***
Summoned (ego)	13.44	(10.87–16.63) ***	13.44	(10.87–16.63) ***
Previously deposed (ego)	1.88	(1.55–2.28) ***	1.88	(1.55–2.28) ***
Tortured (ego)	2.88	(2.29–3.61) ***	2.88	(2.29–3.61) ***
Log likelihood	−1,421		−1,421	
Akaike Information Criterion (AIC)	2,854		2,854	
Bayesian Information Criterion (BIC)	2,882		2,882	
Number of observations	833		833	
*Choice function*				
Woman (alter)	1.17	(0.98–1.40)	1.33	(1.11–1.60) **
Same sex	1.16	(0.98–1.38)	1.13	(0.95–1.34)
Deceased (alter)	0.63	(0.40–0.98) *	0.64	(0.41–1.01)
Reciprocity	4.37	(3.42–5.59) ***	4.42	(3.41–5.73) ***
Common denouncer	1.77	(1.63–1.92) ***	1.67	(1.53–1.81) ***
In-degree popularity (alter) (sqrt)	1.52	(1.39–1.66) ***	1.31	(1.18–1.45) ***
Summoned (alter)			1.79	(1.44–2.22) ***
Absconded from the trial (alter)			1.66	(1.24–2.21) ***
Log likelihood	−4,191		−4,174	
AIC	8,394		8,363	
BIC	8,422		8,401	
Number of observations	829		829	

The table presents the exponentiated coefficients from the DyNAM models along with their 95% CIs and significance levels (*P < 0.05, **P < 0.01, ***P < 0.001). The top panel shows values related to the rate function. The bottom panel displays values related to the choice function. In estimating the rate functions, individuals who never deposed were treated as nonpresent. Thus, our analysis is based on the comparison of the 110 deponents. For estimating the choice function, deponents could potentially select from any of the 239 individuals reported as heretics to the inquisitor. When estimating denunciations directed at family members, the choice set is limited to those identified as family members of the denouncer.

## Conclusion

History is composed of institutions, structures, and narratives, but also of particular situations at particular times, linked in dynamic sequences with a distinct logic of action. An analytical perspective focused on the temporal order of events is useful in understanding both micro-level events, such as the unfolding of an inquisitorial trial, and macro-level processes, such as the evolution of the institution of the Inquisition and its attempts to control religious dissent.

In contrast to exogenous accounts of denunciation articulating opportunities to settle personal scores or incentives associated with institutional regimes (13), we highlighted the role of exposure to pressure as a key antecedent of denunciation. The results of the analysis portrayed the trial as a high-stakes game, with the inquisitor increasing pressure by issuing summonses, calling deponents for a second deposition, and seeking to verify past denunciations. The deponents reacted through coordination with others, through attempts to blur the lines between denouncer and denounced, and the formation of denunciation triads. The identified temporal pattern (Fig. 1) attests that coercive pressure compelled denouncers to implicate individuals socially closer to them and led to behavioral ambiguity. Deponents simultaneously concealed and disclosed, and even when they started to disclose more, they still tried to conceal, holding out on naming kin members until they felt they could do so no longer.

This trial is an illustration of the disheartening fact that even a cohesive community can be fractured with the skillful application of repressive methods using information collected from denunciations. A key finding is that the roles of denouncer and denounced rested with different people at the early stage but became entwined when coercive pressure increased: the denounced was a denouncer too. This is when damage to the social fabric is the deepest, as practically anyone can be a denouncer and suspicion becomes pervasive. The damage endures to this day (33).

However, the extent of the damage was limited by endogenous social forces. It was manifested in the ways in which inferential and/or behavioral mechanisms (29, 30) generated increasing informational redundancies, as deponents denounced others who had already been denounced, summoned or had fled the trial. These denunciations were psychologically “safer” than betraying a fellow Waldensian, who had yet to be identified as one. This dynamic is very important in understanding both the duration of the trials and their broader consequences. Historians tend to attribute the relatively short duration of inquisitorial trials to the difficulties encountered in ensuring the cooperation of local authorities (17, 25), but our results highlight another factor at play: the informational redundancies that a highly focused inquiry (Fig. 2) provoked. It appears that inquisitors pursued quick wins by drilling down a segment of the network, but this tactic constrained their ability to root out religious dissent.

Our findings bring to the fore the possibility of a trade-off between the efficiency of local crackdowns and the effectiveness of curbing the global diffusion of religious dissent. Church authorities were aware that heretic beliefs traveled in the family (34), elaborating interrogative methods to fracture family-based forms of solidarity. The social organization of local populations, however, constrained their utility. To prevent the global diffusion of dissent required reinforcement of coercive pressure and expansion of the scope of control, in ways that the medieval Inquisition was organizationally ill-equipped to achieve (17, 25). It was relatively efficient in implementing local crackdowns but could not muster sufficient resources for a sustained campaign against an adversary adapting to its operations. We encourage more attention to the trade-offs in pursuing local versus more global forms of control.

Our analysis is based on archival data from one historical location, but single cases are sociologically valuable when permitting to extrapolate a generalizable tendency and observe the operation of social mechanisms in a detailed manner rarely afforded by large samples (35). The Giaveno trial was not exceptional in terms of its organization, duration or data availability: it shares many features with trials from the period (see SI Appendix Section D). Our results attest to the fundamental role of coercive pressure in asserting social control by encouraging denunciations: a dynamic readily observable in more recent historical events, such as the Spanish Post-Civil War Repression (36) or the “Great Terror” in Russia (11) (see SI Appendix Section D). The medieval Catholic Church created an organizational template for the systematic suppression and control of difference that political ideology and technological innovation in subsequent centuries would ultimately bring to fruition.

## Materials and methods

### Data

We constructed the variables by manually coding information from the trial proceedings. De Castellario interrogated 110 individuals, and a total of 267 people were either deponents, suspects, or both. Depositions were dated, allowing us to conceptualize the response variable as a relational event connecting each denouncer (*i*) to the person(s) denounced (*j*) on the day of the deposition (*t*): $d_{i\to j,t}$. When a denunciation targeted a household instead of an individual, all household members were considered as targets. The register comprises 829 denunciations, with 753 (90.8%) stating the name of the target.

Most variables are time-varying. These include the number of denunciations received by a person, whether the inquisitor summoned the person, whether the person has been interrogated previously (20 individuals were deposed on two occasions), and whether the person has been tortured. We have precise dates for when people were deposed and denounced, allowing to determine whether a name was mentioned for the first, second, or subsequent time. For summonses, we used either the dates of the four public calls issued by the inquisitor (see Fig. 1) or the day before, in cases when a deposition mentioned a summons not featured in those calls.

We used all available information to identify the relationship between a deponent and the person he or she denounced. Denunciations could target a kin member (80 instances), another member of the heretic congregation (537 instances), or a fellow villager. A kin member could be a household member or extended family, such as grandparents, grandchildren, cognates, or affines. A denunciation targeting a congregation member was inferred from statements in the register indicating association, such as accompanying each other to a meeting with a Waldensian master. This association could be inferred from testimony by one or both parties or by a third person.

Individual-level information such as age or profession is mostly missing. We know the sex of individuals and whether the denounced person was deceased at the time of the trial. Five individuals were summoned but decided to leave the village, never appearing before the inquisitor; they were categorized as “absconded from the trial.” Further details regarding descriptive information and operationalization are given in SI Appendix Section A.

### Statistical procedure

To gain insights into the mechanisms underlying our denunciation network, we estimated a dynamic network actor model (DyNAM) (19). A DyNAM consists of a two-step process. First, it models “individual activity rates”: the waiting time until an actor *i* becomes active and sends a denunciation. Once a specific actor becomes active, the second step determines the target of the denunciation (*j*). These two steps are assumed to be conditionally independent given the process state (*y*). Formally, our models utilize the following formula:


$$\psi_{ij}(y|\theta ,\beta )=\tau_{i}(y|\theta )\times p(d_{i\to j}|y,\beta ).$$

The waiting time is modeled as a composite Poisson process, where each possible denunciation ($d_{i\to j}$) is associated with a Poisson rate $\psi_{ij}(y|\theta ,\beta )$. The waiting time $\tau_{i}\;$ of actor *i* is modeled using an exponential link function:


$$\tau_{i}(y|\theta )=\exp\left(\theta_{0}+\underset{k}{\sum }\;\theta_{k}r_{k}(i,y)\right),$$

where $\theta_{0}$ represents the general tendency of each actor to send denunciations over time, serving as the intercept, and *θ* is the parameter vector associated with r(i,y), which accounts for differences between actors regarding their attributes or positions in the process state. This includes the sex of *i*, the number of denunciations received (in-degree activity), whether *i* has been summoned, whether *i* has been subjected to a previous interrogation, and whether *i* has been tortured.

Once an actor *i* becomes active, the probability that they denounce another actor *j* is modeled using a multinomial probability distribution, dependent on the process state *y* and the parameter vector *β*. Following the logic of a stochastic actor-oriented model (37), the second step exhibits an actor-oriented nature. Namely, ties are weighted against one another by the sending actor *i*, and tie positions depend on which actor sends the tie. Formally, this “choice function” is expressed with the following formula:


$$p(d_{i\to j}|y,\beta )=\frac{\exp(\beta^{T}s(i,j,y))}{\sum_{k\in A\setminus \{i\}}\;\exp(\beta^{T}s(i,j,y))},$$

where statistics s(i,j,y) are functions of the process state characterizing the possible event $d_{i\to j}$. In our case, these encompass attributes of actor *j*, such as gender and deceased status; and endogenous processes, including whether *j* has previously denounced the sending actor *i* (reciprocity), and whether both *i* and *j* have been reported by a third party (common sender), and whether *j* is perceived as an attractive target, having been reported by many others before (in-degree popularity). In additional analyses (see SI Appendix Section C), we explore whether actor *j* held a prominent role in the heretic network, was summoned to appear before the inquisitor, or had absconded from the trial (see also Table 1).

The analyses were performed using the R package goldfish 1.6.8. In estimating activity rates, individuals who never deposed were regarded as nonpresent. Thus, our *θ* is based on the comparison of the 110 deponents in the trial. Conversely, for the estimation of the choice function, we utilized the sample of all the individuals eventually reported as heretics (*N* = 239). Despite the relatively large scale of the network, it is common for residents of medieval villages to be acquainted with one another or, at least, be aware of each other. Accordingly, our models incorporate the assumption that each deponent could potentially implicate any member of the network. For estimating denunciations directed at family members, the choice set of individuals potentially denounceable is further restricted to only those identified as family members of the denouncer. Details on model specification are provided in SI Appendix Section B. To facilitate interpretability, values are expressed as ORs by exponentiating the coefficients and their 95% CI.

## Supplementary Material

pgae340_Supplementary_Data

## Data Availability

The data and code required to reproduce all analyses and visualizations in this study can be accessed here: https://github.com/joseluisesna/Denunciations_in_Giaveno_1335.
