# Supplementary material for: Endogenous dynamics of denunciation: Evidence from an inquisitorial trial
Source: PNAS Nexus. 2024 Aug 21;3(9):pgae340. doi: 10.1093/pnasnexus/pgae340 (PMC11378076; doi:10.1093/pnasnexus/pgae340)
Supplement: pgae340_Supplementary_Data [file pgae340_supplementary_data.docx]

**Supporting Information Text**

**Section A. Variable construction and operationalization**

The sample for the analysis comprises 267 individuals: 110 deponents and 239 denounced (with 82 deponents themselves denounced). Waldensian masters from other regions were not considered (16 individuals in total), as the inquisitor was preoccupied with local residents exposed to their preaching. If a denunciation targeted a household rather than a specific individual, we attributed the denunciation to all identifiable members of that household. As all depositions are dated, we have dyad-time observations (e.g., $i$ denounced $j$ on day $t$). Our dataset contains in total 829 denunciations (823 unique denunciations, as 6 dyads are repeated, and 747 if we count only those where the target is explicitly named). The denunciations constitute our dependent variable.

We constructed our variables by coding information from the registers. The sample comprises 156 men (58.4%) and 111 women (41.6%). Among deponents, the proportion of women is smaller: 34 out of 110 (30.9%). Explicit mentions of profession or place of residence were rare in the registers, suggesting that the majority were locals engaged in manual labor. We have information on the profession of 19 individuals (7.1%) and some details on the place of origin or residence of 120 individuals (44.9%). We do not include these variables in the main analysis due to substantial missing information. This is not a likely source of bias, as Waldensian teachings emphasized apostolic poverty, appealing primarily to peasants and laborers (38). Significant unobserved wealth disparities are unlikely to influence the observed patterns.

The next set of variables are time-varying, capturing coercive pressure. The degree of pressure on deponents increases with the number of denunciations received, being summoned, undergoing a second deposition, and being tortured. "In-degree activity" represents the number of denunciations an individual received. For the descriptives, we use the in-degree at the end of the trial. For the analyses, we used the in-degree measured at the time of the deposition. We applied a square root transformation to the measure to account for decreasing marginal contributions: the fact that the difference between having received 1 vs. 0 denunciations is more substantial than that between 7 vs. 6 denunciations.

A person is categorized as "Summoned" after his or her name appears in one of the four public calls issued by the inquisitor or if a deposition explicitly mentions their summons. Overall, 85 out of 110 deponents (77.3%) were summoned. For those summoned in a public call (30 individuals), we have the exact date. It took 0.64 days on average for a deponent to appear before the inquisitor after being summoned, indicating a short spell between summons and deposition. When the exact date of the summons was unknown, we coded it as occurring on the day before the deposition (a one-day lag). "Previously deposed" is an actor-level indicator for whether a person is one of the 20 deponents interrogated on a second occasion. Four individuals were tortured during the trial: Iohannes Gauterii on February 4^th^ and 5^th^, Stephanus Vet on February 6^th^ and 7^th^, Bernardus de Rosseto on February 7^th^, and Iohannes Martini on February 18^th^.^[[1]](#footnote-1)^ As with the summons, we applied a one-day lag to both "Previously deposed" and "Tortured" to capture the effects of these factors on the propensity to denounce.

Upon examining inconsistencies between the public summonses and the names of those deposed, we discovered that five individuals had fled the village, never appearing before the inquisitor. They were Villelmus de Oddo and Petrus de Rosetto (brother of Bernardus de Rosetto), both summoned for the first time on February 2^nd^, Marguerita Borsseta and her daughter, Iohanna, and Marguerita de Prato Ialato, all three summoned on February 19^th^. These five individuals were classified as "absconded from the trial." As the date of their departure is unknown, but has certainly occurred before their summonses became publicly known, we regarded this as a time-fixed effect.

We used all available information in the register to identify the type of tie between a deponent and the person they denounced. Two types of ties are significant: kin ties and congregation fellows. The kin tie typically features the members of a family or household, such as mother, son, sister, or brother, but we also have other ties, such as grandparent-grandchild, brother/sister-in-law, or cognate/affine. Such ties are not always recorded in the registers. Therefore, we inferred them based on three relations: siblings, parent-child, and spouses. For an ego, we derived the following relations: "(half-)sibling" (sibling of ego’s sibling, or child of ego’s parent), "(step-)parent" (sibling of ego’s child, or child of ego’s spouse), "spouse/partner" (parent of ego’s child), "grandparent" (child of ego’s child), "uncle/aunt" (child of ego’s sibling), "sibling-in-law" (spouse of ego’s sibling), and "parent-in-law" (spouse of ego’s child). In total, we identified 182 kin ties, of which 118 (64.8%) were recorded in the register. 80 (64 if considering only recorded ties) out of 829 denunciations were directed at kin members (9.7% of the total).^[[2]](#footnote-2)^

To capture the informal network of dissident activity, we utilized information from depositions on congregations and their participants. This involved statements such as "I was at X's house and saw Y and Z there" or "I accompanied X and Y to meet with Z." We constructed a bipartite or two-mode network (39) connecting people to congregations. Two individuals were considered congregation fellows if they were reported as being present together at one of these events. Since congregations affirming heretical beliefs were viewed as "criminal activity" by the inquisitor, any person reported as part of a congregation is considered "denounced." The denouncer is typically (but not always) a congregation member. The association between two individuals can be inferred from the testimony of one or both of them or a third person. A total of 1,063 ties were identified, with 537 out of 829 denunciations (64.8%) involving congregation fellows who were not kin members (most kin members were also congregation fellows).

Descriptive statistics of the denunciation network are presented in Table S1. The density is understandably low (2.8%), considering the network's size and the sensitive nature of the tie. The proportion of reciprocal ties, where two deponents denounce each other, is relatively high (42.5%). Transitivity (24.7%) indicates a degree of clustering among the deponents. Regarding the origin of denunciations, 81.8% (673 denunciations) were reported by an individual already denounced by the time of their testimony, 85.2% (701 denunciations) were reported by somebody summoned to appear before the inquisitor, and 26.4% (217 denunciations) by an individual in their second deposition. Women sent 195 (23.7%) of the denunciations and received 293 (35.6%) of them. 479 (58.2%) of all denunciations involve individuals of the same sex. Newman's assortativity coefficient (40) (a measure of homophily) is -0.002, suggesting random mixing. A closer analysis reveals that men exhibited greater homophily in their accusations, directing 64.8% of their denunciations towards other men, compared to women who directed only 36.9% of their accusations towards other women. This is supported by both Coleman’s and Segregation Matrix indices, which show positive values for men (Coleman’s index = 0.157; SMI = 0.406) and negative values for women (Coleman’s index = -0.107; SMI = -0.093) (41). Out of all denunciations, 2.4% (20 denunciations) were directed at deceased individuals, and 10.9% (90 denunciations) targeted individuals who had absconded from the trial.

[Table S1 here]

**Section B. Model construction**

We started by examining the 130 depositions documented in the register. Out of the 110 individuals who faced the inquisitor (with 20 appearing twice), 92 (83.6%) implicated at least one person. 60 (54.5%) of them denounced a congregation fellow, while 39 (35.4%) a family member (of which 37 a living kin member). In total, 829 denunciations were recorded (or 823, if we consider 6 repeated names provided by Iohannes Gauterii in his redeposition). Of these, 80 were directed towards kin members, and 537 towards fellow congregants (or 607 if include kin members).

We constructed our models with attention to endogenous effects—those stemming from the denunciation network. By integrating *in-degree activity* into the rate functions, we assessed whether denunciations tended to originate from individuals who had already been reported by others. In the choice function, we included *reciprocity* (whether there existed a propensity for a deponent ($i$) to denounce a person ($j$) who had previously denounced the deponent ($d_{j\to i}$)), *common denouncer* (the inclination for a deponent ($i$) to denounce a person ($j$) who had been previously reported by a third party ($k$) who has also denounced the deponent ($d_{k\to i} \& d_{k\to j}$)), and *in-degree popularity* (the tendency to denounce individuals who have already received numerous denunciations from other deponents). The mathematical formulation of all the effects used in the study is included in Table S2. The results of these models are presented in Table S3.

[Tables S2 and S3 here]

Closer examination of the 130 depositions provided additional insights into the inquisitor’s methods: in 95 cases, the deponent had already been accused by someone else. Depositions from individuals who had been denounced tend to contain more denunciations on average (7.1 vs. 4.3; $t$-test (df = 125.96) = 2.55, $p$ = .012) and are more likely to implicate both congregation fellows (61.1% vs. 11.4%; $\chi^{2}$ (df = 1) = 23.30, $p$ < .001) and kin members (38.9% vs. 5.7%; $\chi^{2}$ (df = 1) = 11.92, $p$ < .001). A similar pattern emerges for the rate of denunciations by individuals who were summoned (6.9 vs. 4.5 on average; $t$-test (df = 118.29) = 2.22, $p$ = .028).

The impact of coercive pressure is manifested in the different behavior of the same individual at different stages of the trial. For example, among the 20 individuals who provided two depositions, the majority (16) refrained from naming anyone during their first deposition. However, a dramatic shift occurred during the second deposition (as depicted in the left section of Fig. S1), where only one person (Iohannes Dudricis) remained silent. The average number of names disclosed during the second deposition was 11.15, more than three times the number during the first deposition (3.15) (bootstrapped mean of the differences = 7.93 [3.35-12.50, 95% CI with 1,000 samples], $p$ = .002). Fig. S1 also shows that only two individuals (Gauterii and Vet, both subjected to torture) provided fewer names during their second deposition compared to their first one.

[Fig. S1 here]

A similar pattern is observed in the central section of Fig. S1, showcasing the denunciations of congregation fellows. Notably, 85% of the redepositions incriminate at least one congregation fellow. As depicted in the right section of Fig. S1, all denunciations of kin members in this group of deponents occurred during the redeposition. Half of all redepositions targeted at least one kin member.

To capture the impact of coercive pressure, we extended the models in Table S3 to incorporate the effects of summonses, redepositions, and being subject to torture on the inclination to denounce. The findings are presented in Table S4. A comparison of the results in Tables S3 and S4 highlights a reverse in sign for the effect of in-degree activity (capturing the impact of the number of denunciations received on the inclination to denounce) following the inclusion of *Summoned (ego),* *Previously deposed (ego)* and *Tortured (ego)*. However, this is not surprising, considering that summonses were typically issued based on prior denunciations.

[Table S4 here]

Due to the small number of torture cases (4), we considered excluding this variable from our analyses. However, including torture helped control for and more accurately estimate the effects of the other two measures of coercive pressure ("Summoned (ego)" and "Previously deposed (ego)"). Results confirm that a person subjected to torture was highly likely to denounce others. This pattern does not apply to kin members, although the insufficient number of cases (only de Rosseto, among the four tortured individuals, mentioned two kin members) may prevent an accurate estimation of this effect.

Finally, we added to the models the limited available information on individual traits, including the sex of the deponent, the sex of the persons denounced, and whether the person denounced was alive or dead. The results are presented in Table S5 (reported as odds ratios in the main text (Fig. 4)). Previous effects remained consistent, but the coefficients revealed that denunciations were more likely to originate from women than men, provided they were subjected to similar levels of coercive pressure. It appears, however that both gender were as frequently chosen as targets. The positive effect of "Same gender" in the middle model reveals some gender homophily, but this only holds for denunciations among congregation fellows. The negative effect for *Deceased (alter)* indicates that naming deceased individuals was infrequent. This may have been influenced by the inquisitor's preference for collecting names of individuals who could be summoned to testify.

[Table S5 here]

No outliers were detected in any of the three models (for denunciations, regardless of targets, directed at congregation fellows, or kin members). An examination of the change points reveals alterations in the rate functions on February 4^th^ (coinciding with Guaterii’s confession under torture), 11^th^ (no later after Goytrati’s redeposition), 14^th^ and 21^st^ (see Fig. S2, Panel A). Change points in the choice functions were identified on February 10^th^, 14^th^ and 21^st^ (see Fig. S2, Panel B). Similar observations apply to the denunciations directed at congregation fellows (Fig. S2, Panels C and D). For observations directed at kin members, no change points were detected in either the rate or choice functions.

[Fig. S2 here]

**Section C. Disentangling the ‘pile on’ effect**

Results (see *SI Appendix* Section B) demonstrate a pattern of preferential attachment (20), with denunciations concentrating around individuals already targeted by numerous others. Out of 239 individuals reported to the inquisitor, 77 received three or more denunciations. Among those most often targeted were Palmerius Goytrati (24 denunciations), Petrus de Rosetto (23 denunciations), and Iohannes Gauterii (22 denunciations). But the individual with the highest number of denunciations (44) was Villelmus de Oddo.

[Table S6 here]

One plausible explanation for their prominence as targets throughout the trial is their influential role within the heretic network. To capture this role, we calculated the eigenvector centrality (31) in the network of congregation fellow ties (see *SI Appendix* Section A) for each individual in the trial. Adding this measure to the choice function of our DyNAM model confirmed that prominence in the informal network predicted becoming a denunciation target (Model 2 in Table S6).

[Fig. S3 here]

The addition of eigenvector centrality significantly enhanced the model fit and negated the effect of in-degree popularity. This is expected, as these two measures are highly correlated (see Fig. S3), given that prominence in the heretic network is established on the basis of associations reported to the inquisitor.

Another explanation for the concentration of blame on certain individuals may have to do with interconnected responses to the actions of the inquisitor. It is reasonable to expect that citizens of Giaveno may have used information from public summonses to concentrate blame on designated individuals. To test this hypothesis, we added Being Summoned to the choice function.^[[3]](#footnote-3)^ Model 3 on Table S6 corroborates the inclination of deponents to report those who have already been summoned, even after controlling for their previous number of denunciations.

People responded in coordinated manner to the actions of the inquisitor, as well as to those of their peers. Among the individuals with the most accusations against them, a few appear to have decided to leave the village, including Villelmus de Oddo, Petrus de Rosetto, and Marguerita Borsseta. We added a variable for individuals who absconded from the trial to the choice function. Model 4 in Table S6 attests that those who absconded were significantly more likely to be denounced, even after controlling for previous denunciations and for public summonses by the inquisitor. The concentration of blame was a consequence of adaptation to the actions of the inquisitor and fellow villagers, likely driven by the desire to spare others.

**Section D. Generalizability and Selection Bias**

The medieval Inquisition was not a centralized institution. The Church fell short of regulating every aspect of the procedure, but inquisitorial trials shared several important characteristics. The application of the inquisitorial process was one of them (23). Aspects of the inquisitors’ legal capacities and their collaboration with ecclesiastical and civil structures were defined in papal decrees. Instructions on the conduct of inquisition, outlined in inquisitorial manuals, were disseminated widely and implemented with fidelity. The manuals provided guidance on the conduct of summonses as one of the three central tasks of the procedure (with the other two being examination and sentencing) (42). Summonses were defined for various categories of suspects and were meant to be announced publicly (43, 44). Inquisitors from Italy (28), England (45), and what is present-day Poland (46), relied on citations not only as a means of delivering suspects but also as a medium of communication, intended to publicly demonstrate the dangers of heresy. The objective of reaching as large an audience as possible (28, 45, 46), was partly responsible for the increasing informational redundancies. The manuals instructed inquisitors to first and foremost obtain the names of suspects from deponents (42–44), who likely knew not only who had already been summoned but also who had repeatedly refused to heed the summonses.

The inquisitorial procedure adopted by the Church for its courts and anti-heretical trials was quickly introduced into the civil judiciary, particularly in Italy (23, 47). Despite the differences in jurisdictions, the procedures applied by inquisitors and civil tribunals had much in common; both considered accusations and citations as a matter of public interest, to be announced by public officials or at parish churches. This applied particularly to contumacious suspects and to cases of peremptory citations (28, 43–46, 48). Thus, citations were an important instrument of pressure on suspects and their social contacts, announcing prosecution and inviting further denunciations.

Throughout medieval Europe, inquisitors were well aware of the benefits of applying pressure on potential witnesses, but also moderating the pressure in increments proportional to the degree of resistance (17, 18). This was combined with the accumulation of incriminating evidence and with citations based on this iterative process. For example, the 13^th^- and 14^th^-century trials in Turin (49, 50), Carcassonne (51), Pamiers (52–54), Toulouse (55, 56) or Szczecin (57) resembled the trial in Giaveno in collecting information by infiltrating the local networks and in applying pressure on unforthcoming witnesses in the form of (repeated) summonses, incarceration, or torture.

The observed application of coercive pressure for denunciation and the resultant informational redundancies can be identified in more recent historical events, such as the Spanish Post-Civil War Francoist Repression. As Anderson (36) remarks, “Francoists strongly encouraged, if not forced, some people to make denunciations” (p. 21). Local officials believed that a wide range of denunciations and denouncers strengthened the prosecution’s case and were keen to collect multiple denunciations against suspected Republicans. The author finds that 27 of the 52 denunciations he studied were made against people whom the authorities already held in custody and whom officials themselves could have denounced (p. 21). Some officials restricted their reports to mere repetitions of what denouncers had already reported. This is consistent with the patterns we identified in Giaveno.

Historians know relatively little about the survival rates of medieval inquisitorial registers. Yet, there is evidence suggesting that documents have been lost over time. This can be due to accidental damage, to the actions of targeted populations or the negligence of officials. For the case of Northern Italy, it has been suggested that the suppression of the Inquisition during the Napoleonic administration may have contributed to the loss or misplacement of documents (58).

Records of the inquisition are housed in archives belonging to the mendicant orders, bishoprics, the papal or civil administration. Historians have yet to investigate in detail these repositories. In general, records were diligently collected and stored, classified in a manner that resembles the categorization used in Giaveno: depositions, abjurations, summonses, etc. These documents were either bounded in separate codices or, as in Giaveno, copied into a single manuscript.

It is unlikely that the Giaveno register is exceptional, as it is similar in key respects to numerous contemporaneous records from France, Germany, and Italy that reveal the same structure, reproducing procedures outlined in inquisitorial manuals. The record is also unexceptional in terms of its size. It is more voluminous than some contemporaneous records, such as those from Szczecin, but its number of documents is far overshadowed by other registers, such as those from Bologna (59) or the famous manuscript 609 of the Bibliothèque de Toulouse (26). This leads us to believe that there is no significant survival bias that may be affecting the results.

**Figures**

**
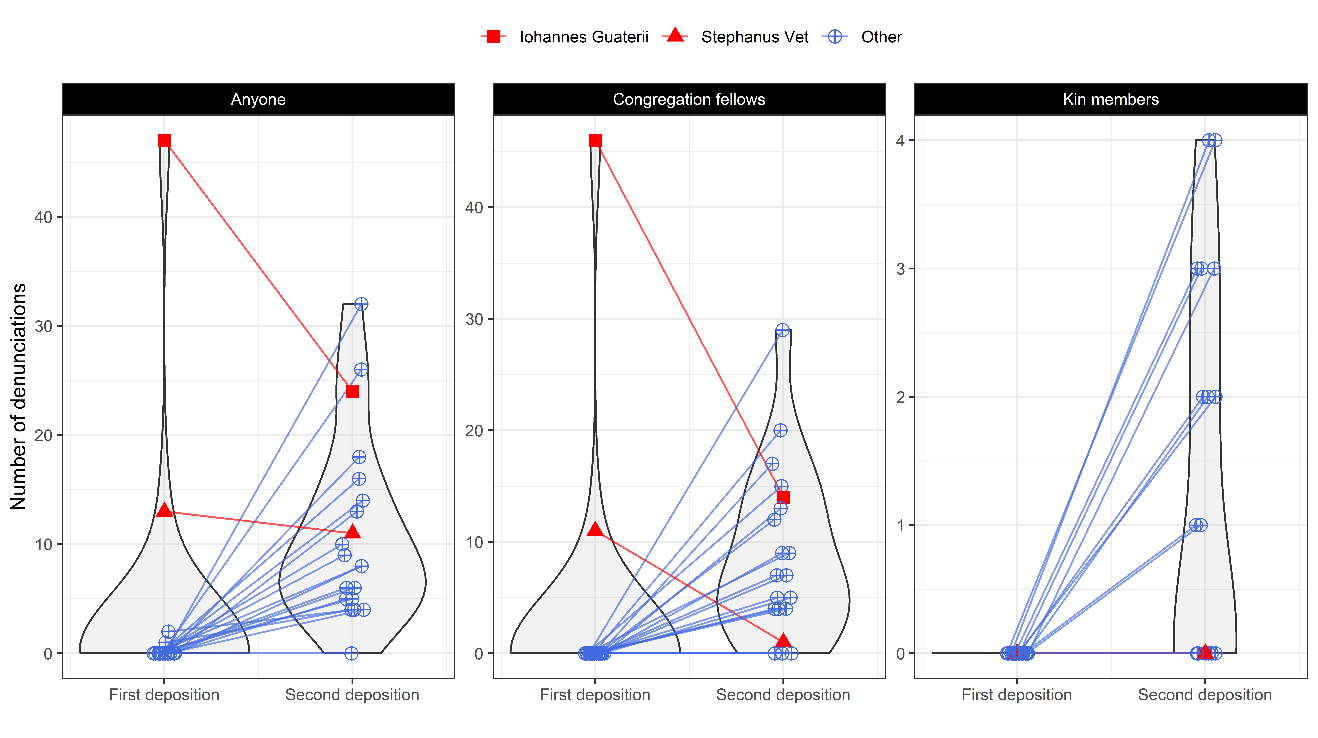
**

**Fig. S1.** Number of denunciations reported to the inquisitor at first vs. second deposition for the 20 individuals deposed in two occasions.

**
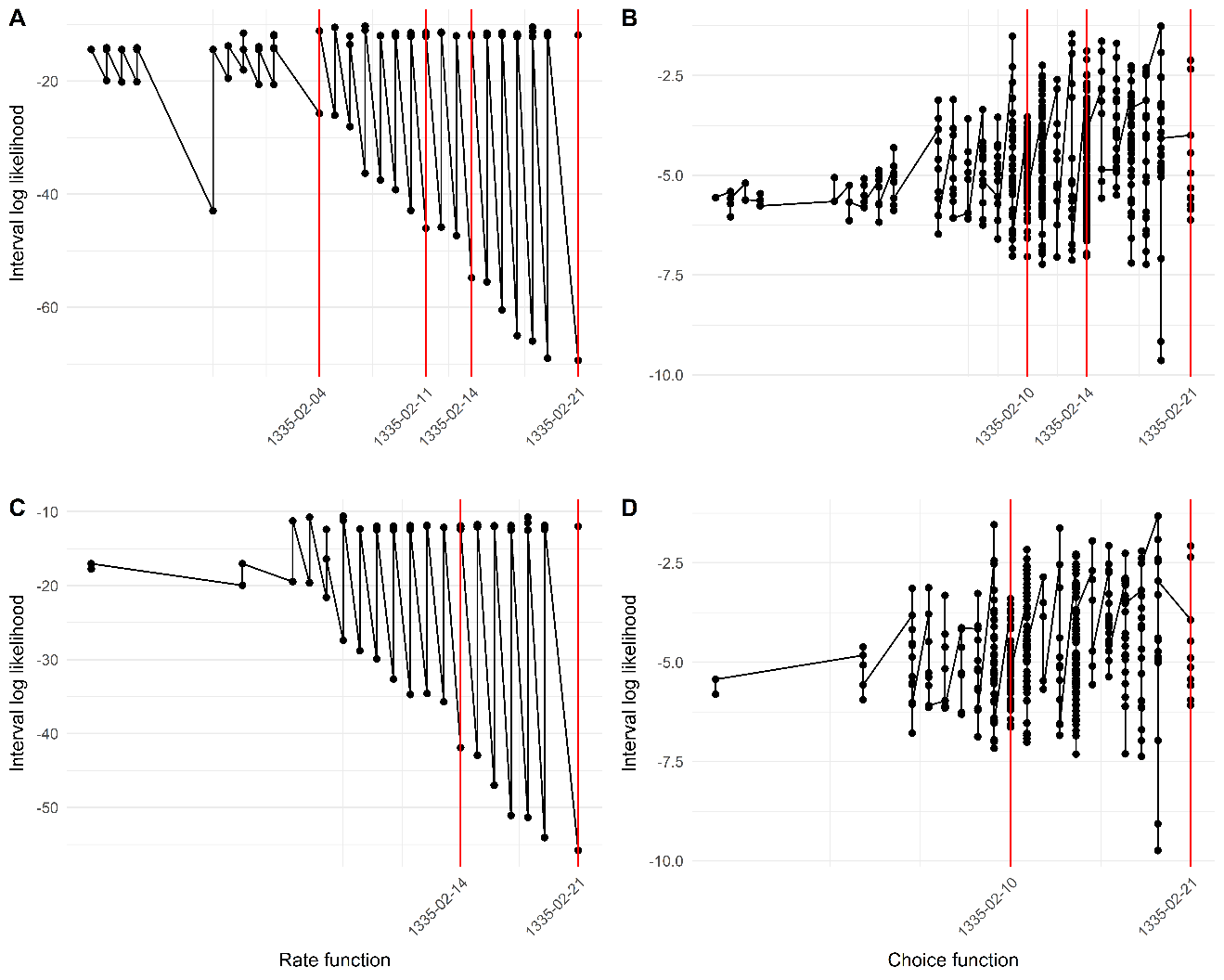
**

**Fig. S2.** Change points in the rate (left) and choice (right) functions for all denunciations (first row), and denunciations at congregation fellows (second row). No change points in either the rate or choice functions were detected for denunciations directed at kin members.

**
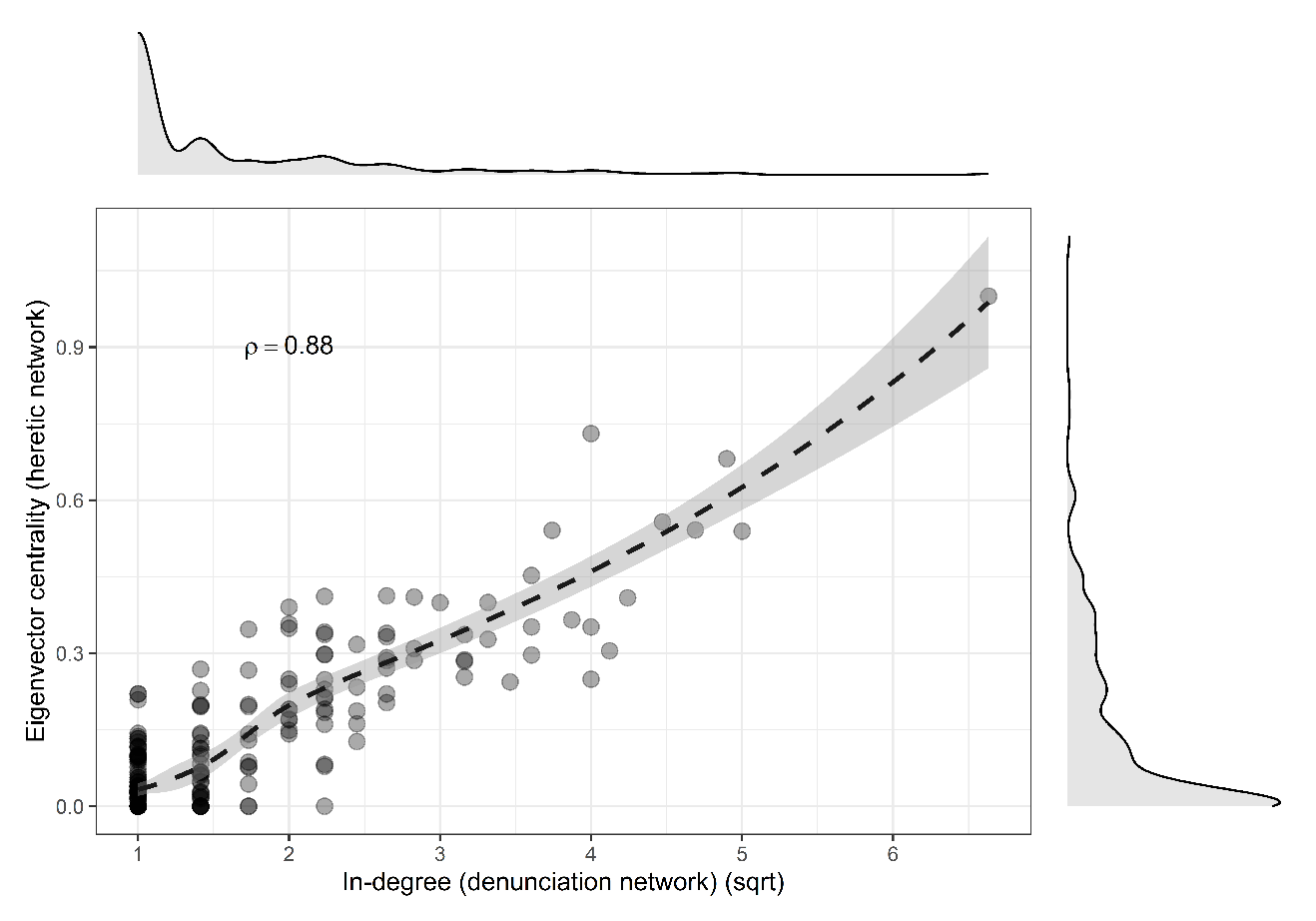
**

**Fig. S3.** Association between in-degree in the denunciation network by the trial’ end and eigenvector centrality in the heretic network

**Table S1.** Descriptives for the variables in the analysis.

|  | Mean | SD | Min | Max |
| --- | --- | --- | --- | --- |
| *All sample (N=267)* |  |  |  |  |
| Woman | 0.42 | 0.49 | 0 | 1 |
| Deceased | 0.07 | 0.26 | 0 | 1 |
| Denunciations received | 3.08 | 4.81 | 0 | 44 |
| Summoned | 0.34 | 0.47 | 0 | 1 |
| Deposed | 0.41 | 0.49 | 0 | 1 |
| Deposed in two occasions | 0.07 | 0.26 | 0 | 1 |
| Tortured | 0.01 | 0.12 | 0 | 1 |
|  |  |  |  |  |
| *Deponents (N=110)* |  |  |  |  |
| Woman | 0.31 | 0.46 | 0 | 1 |
| Denunciations received | 4.36 | 5.30 | 0 | 24 |
| Summoned | 0.77 | 0.42 | 0 | 1 |
| Deposed in two occasions | 0.18 | 0.39 | 0 | 1 |
| Tortured | 0.04 | 0.19 | 0 | 1 |
|  |  |  |  |  |
| *Denunciations (N=823)* |  |  |  |  |
| Density | 0.03 |  |  |  |
| Reciprocity | 0.43 |  |  |  |
| Transitivity | 0.25 |  |  |  |
| To congregation fellow | 0.74 | 0.44 | 0 | 1 |
| To kin | 0.10 | 0.30 | 0 | 1 |
| Previously denounced (denouncer) | 0.82 | 0.39 | 0 | 1 |
| Summoned (denouncer) | 0.85 | 0.36 | 0 | 1 |
| Previously deposed (denouncer) | 0.26 | 0.44 | 0 | 1 |
| Tortured (denouncer) | 0.11 | 0.31 | 0 | 1 |
| Woman (denouncer) | 0.24 | 0.43 | 0 | 1 |
| Woman (target) | 0.36 | 0.48 | 0 | 1 |
| Same sex | 0.58 | 0.49 | 0 | 1 |
| Deceased (target) | 0.02 | 0.15 | 0 | 1 |
| Absconded from the trial (target) | 0.11 | 0.31 | 0 | 1 |
| Note: Density and reciprocity are calculated considering the out-going ties of non-deponents as missing. As zeroes, these values are 0.01 and 0.25, respectively. | | | | |

**Table S2**. DyNAM effects used in the study.

| Name | Mathematical formulation | Goldfish specification | Brief explanation |
| --- | --- | --- | --- |
| ***Rate function*** | | | |
| Indegree | $r\left( i,t,d \right)=\sum_{k} I({d\left( t \right)}_{ki}>0)$ | indeg(type = “ego”, transformFun = sqrt) | Tendency of actor $i$ to denounce when $i$ has a high incoming degree in the denunciation network ($d$) |
| Ego effect | $r\left( i,t,z \right)={z(t)}_{i}$ | ego() | Tendency of actors to be more active when they posses (or score high) on an attribute ($z$) |
| ***Choice function*** | | | |
| Reciprocity | $s\left( i,j,t,d \right)=I({d\left( t \right)}_{ji}>0)$ | recip() | Tendency of $i$ to denounce $j$if $d_{j\to i}$happened before |
| Common sender | $s\left( i,j,t,d \right)=\sum_{k} I({d\left( t \right)}_{ki}>0)I({d\left( t \right)}_{kj}>0)$ | commonSender() | Tendency of $i$ to denounce $j$ when $d_{i\to j}$ closes more two-paths ($i\leftarrow k\to j$) in past denunciations |
| Indegree (alter) | $s\left( i,j,t,d \right)=\sum_{k} I({d\left( t \right)}_{kj}>0)$ | indeg(type = “alter”, transformFun = sqrt) | Tendency to denounce when $j$ has a high incoming degree in the denunciation network ($d$) |
| Alter effect | $s\left( i,j,t,z \right)=z({t)}_{j}$ | alter() | Tendency to denounce when $j$ possess (or scores) high on an attribute ($z$) |
| Same effect | $s\left( i,j,t,z \right)=I({z\left( t \right)}_{i}={z\left( t \right)}_{j})$ | same() | Tendency of a denunciation to happen if actors $i$ and $j$ possess the same attribute value ($z$) |
| Note: ${d(t)}_{ij}$ denotes the value of the denunciation network $d$ at time $t$ between actor $i$ and actor $j$. ${z(t)}_{i}$ denotes the value of an actor attribute $z$ at time $t$ for actor $i$. $I(y)$ denotes the indicator function. It takes the value 1 when variable $y$ is true, zero otherwise. | | | |

**Table S3.** DyNAM estimates (endogenous effects only).

|  | Denunciations  (anyone) | | | Denunciations  (congregation fellows) | | | Denunciations  (kin members) | | |
| --- | --- | --- | --- | --- | --- | --- | --- | --- | --- |
|  | Est. | SE |  | Est. | SE |  | Est. | SE |  |
| ***Rate function*** | | | | | | | | | |
| Intercept | -2.09 | 0.05 | *** | -3.67 | 0.06 | *** | -5.59 | 0.17 | *** |
| In-degree activity (ego) (sqrt) | 0.59 | 0.03 | *** | 0.80 | 0.03 | *** | 0.81 | 0.07 | *** |
| *Log likelihood* | *-1,796* |  |  | *-1,715* |  |  | *-406* |  |  |
| *AIC* | *3,595* |  |  | *3,434* |  |  | *817* |  |  |
| *BIC* | *3,605* |  |  | *3,443* |  |  | *822* |  |  |
| *Number of observations* | *829* |  |  | *543* |  |  | *92* |  |  |
| ***Choice function*** |  |  |  |  |  |  |  |  |  |
| Reciprocity | 1.46 | 0.12 | *** | 1.55 | 0.14 | *** | 0.67 | 0.41 |  |
| Common denouncer | 0.57 | 0.04 | *** | 0.48 | 0.05 | *** | 0.22 | 0.16 |  |
| In-degree popularity (alter) (sqrt) | 0.42 | 0.04 | *** | 0.50 | 0.05 | *** | 0.07 | 0.22 |  |
| *Log likelihood* | *-4,195* |  |  | *-2,664* |  |  | *-93* |  |  |
| *AIC* | *8,396* |  |  | *5,335* |  |  | *193* |  |  |
| *BIC* | *8,410* |  |  | *5,348* |  |  | *200* |  |  |
| *Number of observations* | *829* |  |  | *537* |  |  | *80* |  |  |
| Note: The table presents the DyNAM estimates along with their standard errors and significance levels (* p < .05, ** p < .01, *** p < .001). The top panel shows values related to the rate function. The bottom panel displays values related to the choice function. In estimating the rate functions, individuals who never deposed were treated as non-present. Thus, our analysis is based on the comparison of the 110 deponents. For estimating the choice function, deponents could potentially select from any of the 239 individuals reported as heretics to the inquisitor. When estimating denunciations directed at family members, the choice set is limited to those identified as family members of the denouncer. | | | | | | | | | |

**Table S4.** DyNAM estimates (endogenous effects plus inquisitor's actions).

|  | Denunciations  (anyone) | | | Denunciations  (congregation fellows) | | | Denunciations  (kin members) | | |
| --- | --- | --- | --- | --- | --- | --- | --- | --- | --- |
|  | Est. | SE |  | Est. | SE |  | Est. | SE |  |
| ***Rate function*** | | | | | | | | | |
| Intercept | -2.93 | 0.09 | *** | -5.91 | 0.22 | *** | -8.90 | 1.00 | *** |
| In-degree activity (ego) (sqrt) | -0.14 | 0.04 | *** | -0.11 | 0.04 | ** | -0.14 | 0.11 |  |
| Summoned (ego) | 2.55 | 0.11 | *** | 5.16 | 0.23 | *** | 6.33 | 1.02 | *** |
| Previously deposed (ego) | 0.57 | 0.10 | *** | 0.40 | 0.11 | *** | 1.05 | 0.27 | *** |
| Tortured (ego) | 1.03 | 0.12 | *** | 1.19 | 0.13 | *** | -1.27 | 0.73 |  |
| *Log likelihood* | *-1,427* |  |  | *-979* |  |  | *-288* |  |  |
| *AIC* | *2,863* |  |  | *1,968* |  |  | *586* |  |  |
| *BIC* | *2,887* |  |  | *1,990* |  |  | *599* |  |  |
| *Number of observations* | *833* |  |  | *547* |  |  | 96 |  |  |
| ***Choice function*** | | | | | | | | | |
| Reciprocity | 1.46 | 0.12 | *** | 1.55 | 0.14 | *** | 0.67 | 0.41 |  |
| Common denouncer | 0.57 | 0.04 | *** | 0.48 | 0.05 | *** | 0.22 | 0.16 |  |
| In-degree popularity (alter) (sqrt) | 0.42 | 0.04 | *** | 0.50 | 0.05 | *** | 0.07 | 0.22 |  |
| *Log likelihood* | *-4,195* |  |  | *-2,664* |  |  | *-93* |  |  |
| *AIC* | *8,396* |  |  | *5,335* |  |  | *193* |  |  |
| *BIC* | *8,410* |  |  | *5,348* |  |  | *200* |  |  |
| *Number of observations* | *829* |  |  | *537* |  |  | *80* |  |  |
| Note: The table presents the DyNAM estimates along with their standard errors and significance levels (* p < .05, ** p < .01, *** p < .001). The top panel shows values related to the rate function. The bottom panel displays values related to the choice function. In estimating the rate functions, individuals who never deposed were treated as non-present. Thus, our analysis is based on the comparison of the 110 deponents. For estimating the choice function, deponents could potentially select from any of the 239 individuals reported as heretics to the inquisitor. When estimating denunciations directed at family members, the choice set is limited to those identified as family members of the denouncer. | | | | | | | | | |

**Table S5.** DyNAM estimates (endogenous effects, inquisitor's actions, and individuals' attributes).

|  | Denunciations  (anyone) | | | Denunciations  (congregation fellows) | | | Denunciations  (kin members) | | |
| --- | --- | --- | --- | --- | --- | --- | --- | --- | --- |
|  | Est. | SE |  | Est. | SE |  | Est. | SE |  |
| ***Rate function*** | | | | | | | | | |
| Intercept | -3.05 | 0.09 | *** | -6.15 | 0.23 | *** | -9.31 | 1.01 | *** |
| Woman (ego) | 0.30 | 0.09 | *** | 0.49 | 0.11 | *** | 0.79 | 0.26 | ** |
| In-degree activity (ego) (sqrt) | -0.15 | 0.04 | *** | -0.11 | 0.04 | ** | -0.15 | 0.11 |  |
| Summoned (ego) | 2.60 | 0.11 | *** | 5.28 | 0.24 | *** | 6.52 | 1.02 | *** |
| Previously deposed (ego) | 0.63 | 0.10 | *** | 0.50 | 0.12 | *** | 1.27 | 0.28 | *** |
| Tortured (ego) | 1.06 | 0.12 | *** | 1.25 | 0.14 | *** | -1.21 | 0.73 |  |
| *Log likelihood* | *-1,421* |  |  | *-969* |  |  | *-284* |  |  |
| *AIC* | *2,854* |  |  | *1,951* |  |  | 579 |  |  |
| *BIC* | *2,882* |  |  | *1,976* |  |  | *595* |  |  |
| *Number of observations* | *833* |  |  | *547* |  |  | *96* |  |  |
| ***Choice function*** | | | | | | | | | |
| Woman (alter) | 0.16 | 0.09 |  | 0.16 | 0.11 |  | 0.81 | 0.47 |  |
| Same sex | 0.15 | 0.09 |  | 0.21 | 0.11 | * | 0.35 | 0.35 |  |
| Deceased (alter) | -0.47 | 0.23 | * | -0.72 | 0.34 | * | 0.83 | 0.75 |  |
| Reciprocity | 1.48 | 0.13 | *** | 1.56 | 0.14 | *** | 1.22 | 0.53 | * |
| Common denouncer | 0.57 | 0.04 | *** | 0.49 | 0.05 | *** | 0.25 | 0.18 |  |
| In-degree popularity (alter) (sqrt) | 0.42 | 0.04 | *** | 0.50 | 0.05 | *** | 0.30 | 0.26 |  |
| *Log likelihood* | *-4,191* |  |  | *-2,660* |  |  | *-91* |  |  |
| *AIC* | *8,394* |  |  | *5,331* |  |  | *194* |  |  |
| *BIC* | *8,422* |  |  | *5,357* |  |  | *208* |  |  |
| *Number of observations* | *829* |  |  | *537* |  |  | *80* |  |  |
| Note: The table presents the DyNAM estimates along with their standard errors and significance levels (* p < .05, ** p < .01, *** p < .001). The top panel shows values related to the rate function. The bottom panel displays values related to the choice function. In estimating the rate functions, individuals who never deposed were treated as non-present. Thus, our analysis is based on the comparison of the 110 deponents. For estimating the choice function, deponents could potentially select from any of the 239 individuals reported as heretics to the inquisitor. When estimating denunciations directed at family members, the choice set is limited to those identified as family members of the denouncer. | | | | | | | | | |

**Table S6.** DyNAM estimates disentangling the ‘pile on’ effect.

|  | Denunciations  (anyone)  (1) | | | Denunciations  (anyone)  (2) | | | Denunciations  (anyone)  (3) | | | Denunciations  (anyone)  (4) | | |
| --- | --- | --- | --- | --- | --- | --- | --- | --- | --- | --- | --- | --- |
|  | Est. | SE |  | Est. | SE |  | Est. | SE |  | Est. | SE |  |
| ***Rate function*** | | | | | | | | | | | | |
| Intercept | -3.05 | 0.09 | *** | -3.05 | 0.09 | *** | -3.05 | 0.09 | *** | -3.05 | 0.09 | *** |
| Woman (ego) | 0.30 | 0.09 | *** | 0.30 | 0.09 | *** | 0.30 | 0.09 | *** | 0.30 | 0.09 | *** |
| In-degree activity (ego) (sqrt) | -0.15 | 0.04 | *** | -0.15 | 0.04 | *** | -0.15 | 0.04 | *** | -0.15 | 0.04 | *** |
| Summoned (ego) | 2.60 | 0.11 | *** | 2.60 | 0.11 | *** | 2.60 | 0.11 | *** | 2.60 | 0.11 | *** |
| Previously deposed (ego) | 0.63 | 0.10 | *** | 0.63 | 0.10 | *** | 0.63 | 0.10 | *** | 0.63 | 0.10 | *** |
| Tortured (ego) | 1.06 | 0.12 | *** | 1.06 | 0.12 | *** | 1.06 | 0.12 | *** | 1.06 | 0.12 | *** |
| *Log likelihood* | *-1,421* |  |  | *-1,421* |  |  | *-1,421* |  |  | *-1,421* |  |  |
| *AIC* | *2,854* |  |  | *2,854* |  |  | *2,854* |  |  | *2,854* |  |  |
| *BIC* | *2,882* |  |  | *2,882* |  |  | *2,882* |  |  | *2,882* |  |  |
| *Number of observations* | *833* |  |  | *833* |  |  | *833* |  |  | *833* |  |  |
| ***Choice function*** | | | | | | | | | | | | |
| Woman (alter) | 0.16 | 0.09 |  | 0.27 | 0.09 | ** | 0.28 | 0.09 | ** | 0.29 | 0.09 | ** |
| Same sex | 0.15 | 0.09 |  | 0.11 | 0.09 |  | 0.12 | 0.09 |  | 0.12 | 0.09 |  |
| Deceased (alter) | -0.47 | 0.23 | * | -0.26 | 0.23 |  | -0.44 | 0.23 |  | -0.44 | 0.23 |  |
| Reciprocity | 1.48 | 0.13 | *** | 1.48 | 0.12 | *** | 1.35 | 0.13 | *** | 1.49 | 0.13 | *** |
| Common denouncer | 0.57 | 0.04 | *** | 0.46 | 0.04 | *** | 0.54 | 0.04 | *** | 0.51 | 0.04 | *** |
| In-degree popularity (alter) (sqrt) | 0.42 | 0.04 | *** | -0.04 | 0.05 |  | 0.33 | 0.05 | *** | 0.27 | 0.05 | *** |
| Eigenvector centrality in heretic network (alter) |  |  |  | 3.07 | 0.19 | *** |  |  |  |  |  |  |
| Summoned (alter) |  |  |  |  |  |  | 0.54 | 0.11 | *** | 0.58 | 0.11 | *** |
| Absconded from the trial (alter) |  |  |  |  |  |  |  |  |  | 0.51 | 0.15 | *** |
| *Log likelihood* | *-4,191* |  |  | *-4,078* |  |  | *-4,179* |  |  | *-4,174* |  |  |
| *AIC* | *8,394* |  |  | *8,170* |  |  | *8,372* |  |  | *8,363* |  |  |
| *BIC* | *8,422* |  |  | *8,203* |  |  | *8,405* |  |  | *8,401* |  |  |
| *Number of observations* | *829* |  |  | *829* |  |  | *829* |  |  | *829* |  |  |
| Note: The table presents the DyNAM estimates along with their standard errors and significance levels (* p < .05, ** p < .01, *** p < .001). The top panel shows values related to the rate function. The bottom panel displays values related to the choice function. In estimating the rate functions, individuals who never deposed were treated as non-present. Thus, our analysis is based on the comparison of the 110 deponents. For estimating the choice function, deponents could potentially select from any of the 239 individuals reported as heretics to the inquisitor. When estimating denunciations directed at family members, the choice set is limited to those identified as family members of the denouncer. | | | | | | | | | | | | |

1. Unlike the first three individuals, whose torture was aimed at gaining access to the heretic network, Martini was tortured to elicit confession from someone who had already appeared at the trial and had committed perjury. [↑](#footnote-ref-1)
2. Of the 80 denunciations directed at a kin member, 64 (80.0%) pertain to a member of the immediate (rather than extended) family: a spouse, a sibling, a parent, or a child. The relative paucity of the denunciations prevents further disaggregation, for instance: immediate vs. extended family, matrilineal vs. patrilineal, or ancestor-focus vs. ego-focus (17). [↑](#footnote-ref-2)
3. For the choice function, undated summonses can only affect the decision of subjects deposed on the same or subsequent day to the summoned person, but not before. [↑](#footnote-ref-3)
